# Evaluation of physicochemical and textural properties of myofibrillar protein gels and low-fat model sausage containing various levels of curdlan

**DOI:** 10.5713/ajas.18.0585

**Published:** 2018-11-28

**Authors:** Chang Hoon Lee, Koo Bok Chin

**Affiliations:** 1 Department of Animal Science, Chonnam National University, Gwangju 61186, Korea

**Keywords:** Curdlan, Pork Myofibrillar Protein, Viscosity, Gel Strength, Low-fat Model Sausage

## Abstract

**Objective:**

Curdlan has been widely used as a gelling agent in various food systems. This study was performed to evaluate the rheological properties of pork myofibrillar protein (MP) with different levels of curdlan (0.5% to 1.5%) and its application to low-fat model sausages (LFS).

**Methods:**

MP mixtures were prepared with 0.5%, 1.0%, and 1.5% of curdlan. Cooking loss (%), gel strength (gf), shear stress (Pa), and scanning electron microscopy were measured. Physicochemical and textural properties of LFS containing different levels of curdlan were measured.

**Results:**

The shear stress of MP mixtures increased with increasing levels of curdlan. MP gels with increased levels of curdlan decreased cooking loss and increased gel strength (p<0.05). The MPs with 1.0% and 1.5% of curdlan were observed more compact three-dimensional structure than those with 0.5% curdlan. Increased curdlan level in LFS affected redness (a*) and yellowness (b*) values. Although expressible moisture of LFS did not differ among curdlan levels, LFSs with various levels of curdlan decreased cooking loss as compared to control sausages. Hardness values (2,251 to 2,311 gf) of LFS with 0.5% and 1.0% curdlan was increased and differ from those (1,901 gf) of control sausages.

**Conclusion:**

The addition of 1.0% curdlan improved the functional and textural properties of LFS.

## INTRODUCTION

Myofibrillar protein (MP) is composed of a variety of structural proteins including myosin, actin, tropomyosin, troponin, α-actinin, and desmin [1]. It can be obtained from muscle cells extracted with a high salt solution (>0.6 M). MP has been strongly associated with the binding and water-holding capacity of meat and meat products [2]. In addition, rheological properties of myofibrils are changed by electrostatic repulsion in a pH-dependent fashion. Other factors that influence MP gels are sodium concentration and non-protein polymer ingredients, such as hydrocolloids and fibers [3].

Hydrocolloids including carrageenan, starch, gelatin, and xanthan can functionally improve rheological properties in the food system. They are generally used in comminuted meat products to improve emulsion stability, water/fat binding, and texture, and also can structurally interact with muscle protein. Gelatin can stabilize shrinkage and improve cooking yield due to their gelling and water binding properties [4]. Carrageenan improves the textural properties and appearance of sausages [5]. A study by Ruiz-Capillas et al [6] demonstrated that konjac gel has used as a fat replacer in dry fermented sausages. Lin and Huang [7] reported that konjac/gellan mixed gels were acceptable fat replacers in reduced-fat frankfurters, with a resulting positive sensory score and an adequate shelf life. Chin et al [8] reported that a desirable gel structure was obtained by the combination of casein and soy protein in MP gels under the proper salt and pH conditions. Locust bean gum interacts synergistically with other hydrocolloids and acts as a thickening agent and a stabilizer in food systems [9].

Curdlan is a thermo-gel stable and water-soluble polysaccharide composed of β-(1,3)-linked glucosidic linkages and glucose, galactose, and mannose in an approximate molar ratio of 87.7:11.0:1.3 [10]. It is produced by the microorganism *Alcaligenes faecalis* var. *myxogenes* 10C3. Curdlan is odorless, tasteless, and colorless [11]. When aqueous curdlan solution is heated, its gel strength increases with increasing heating temperature and time [12]. The adhesiveness and viscosity of emulsified meatballs are increased by the addition of curdlan (0.6%) [13], which was effective as fat-replacer in non-fat sausages [14]. Curdlan could influence the quality of meat products by improving the water-entrap and replacing the fat in thermal-irreversible gels [15]. Finally, curdlan (1.0%) needed to improve texture in meat gels [16].

The previous studies reported the benefits of curdlan in meat products. However, the influence of various concentrations of curdlan on quality of meat products has not been thoroughly studied. Thus, this study carried out to determine the optimum content of curdlan on the rheological properties of pork MP gels and application to low-fat model sausages.

## MATERIALS AND METHODS

### Experiment 1: Interaction of MP and curdlan

#### Materials

Slaughtered pork loins (Landrace×Yorkshire, grade A, Korea) on the same day were purchased from a local meat market (Samho Co., Gwangju, Korea). Visible fat and connective tissues were removed. The meat was formed into 1 to 2 cm^3^ cubes and stored in a –50°C freezer until untilized. Curdlan was provided by the Sncfood company (Seoul, Korea).

#### Processing of myofibrillar protein gels

The frozen pork loin was thawed overnight at 4°C. The MP was adjusted to a concentration of 40 mg/mL after extraction for three times using 0.1 M NaCl in 50 mM phosphate buffer by food mixer (Bowl Rest mixer, Hamilton Beach/Proctor-Silex, Inc., Southern Pines, NC, USA) for 1.5 min each time. The resulting emulsion was washed with 0.1 M NaCl buffer using cheesecloth to remove connective tissue [17]. MP, buffer, and curdlan (0.5%, 1.0%, and 1.5%) were mixed in the proportions, and 5 mL of the protein mixture was loaded into vial s which were heated from 20°C to 80°C with an incremental temperature increase of 3°C/min in a water bath (WB-22, Daihan Scientific Co., Seoul, Korea). The cooked MP gels were quickly chilled in an ice water and stored at refrigerator temperature (4°C±2°C) until analyzed.

#### Evaluation of raw or cooked myofibrillar protein gels

Approximately 3 mL of each sample mixture was loaded into the probe container of the concentric cylinder type rotational rheometer (RC30, Rheotec Messtechnik GmbH, Ottendorf-Okrilla, Germany). The shear rate was increased from 0 to 300/s for 60 s. The shear rate was plotted against shear stress using Excel 2016 software (Microsoft Corporation, Redmond, WA, USA). Cooking loss (CL) of MP gels heated as described above was determined as the average of five different samples and calculated as the weight before cooking compared to the weight after cooking.

Gel strength of cooked MP gels was measured by Universal Testing Machine (3344, Instron Corporation, Norwood, MA, USA). The breaking force (gf) of five different samples was determined at a head speed of 500 mm/min by steel drill chuck (33BA 1/2–20, Jacobs chuck, Sparks, MD, USA). The expressed value was the average of the five determinations.

The three-dimensional structures of MP gels were measured by low-vacuum scanning electron microscopy (LV-SEM) (JSM-6610LV, JEOL Ltd., Tokyo, Japan). Cubed samples (3×3×3 mm^3^) were immersed in 2.5% glutaraldehyde (pH 7) and 0.1 M phosphate buffer solution overnight at 4°C and then in osmium tetroxide (pH 7) and 0.1 M phosphate buffer for 5 h. After rinsing three times, each sample was dehydrated by immersion for 10 min in a sequentially increasing series of ethanol solutions (50%, 60%, 70%, 80%, 90%, and 100%). Each sample was completely dried by immersion in acetone for 10 min. The dehydrated and dry samples were each gold-coated using a model 108 auto sputter coater (Cressington Scientific Instruments Ltd., Watford, England) prior to LV-SEM microstructure examination.

#### Statistical analysis

The whole experiment was performed in triplicates. Data were analyzed by one-way analysis of variance (ANOVA) followed by Duncan’s test using SPSS 20.0 statistical software at a significant level of 0.05 (SPSS Inc., Chicago, IL, USA). A p-value <0.05 indicated a significant difference.

### Experiment 2: Quality of low-fat sausage prepared with curdlan

#### Materials

Pork ham (Landrace×Yorkshire, Grade A) was purchased for each replication from the aforementioned local market. All visible fat and connective tissue were removed and the remaining meat was ground using meat chopper (M-12S, Hankook Fujee Industries Co., Ltd., Busan, Korea). Curdlan was obtained from the aforementioned source.

#### Processing of low-fat model sausages

Model sausages were manufactured with 60% pork, 38% water, 1.3% salt, 0.4% sodium tripolyphosphate, 0.05% sodium erythorbate, 0.25% curing salt and respectively 0%, 0.5%, 1%, and 1.5% curdlan as followed by Lee and Chin study [18]. The ground pork ham was mixed with curing ingredients using a cutter (HMC-401, Hanil Electric, Seoul, Korea) for 3 min. The emulsified mixture was stuffed in 45 mL plastic tubes and cooked until reaching 72°C at the geometric center of each sample. Fully cooked samples were kept in an ice water while they were completely cooled and then stored in the refrigerator (4°C±2°C) until used.

#### Evaluations of low-fat model sausages

##### i) Chemical composition, pH, and color

Moisture, fat, and protein contents were measured using AOAC methods [19]. pH was determined five times from randomly selected samples (n = 5) using the pH-meter (MP-120, Mettler-Toledo, Greifeense, Switzerland) with Inlab 413/IP 67 electrode. Color values (Commission Internationale de l’Eclairage [CIE]) lightness [L*], redness [a*], yellowness [b*]) were calculated as the average of six determinations made on the internal surface of samples using the color reader (CR-10, Minolta, Tokyo, Japan). Color reader calibrated with white plate standard (L* = 95.6, a* = 1.0, and b* = 0.2). And it characterizes standard illuminant D_65_ at an observation angle of 8°.

##### ii) Cooking loss (%) and expressible moisture (%)

The CL (%) was calculated as the weight before cooking compared to the weight after cooking. Expressible moisture (EM, %) was evaluated as previously described [20]. Sausage samples (n = 4) were prepared as cubes with an approximate weight of 1.5 g. Prepared samples were wrapped in three pieces of Whatman #3 filter paper and centrifuged at 1,660×g for 15 min in a centrifuge (VS-5500, Vision Science Co., Ltd, Seoul, Korea). The EM was calculated from the weight before and after centrifugation.

##### iii) Textural properties

Sausage samples (n = 10) were shaped 13 mm in height and 12.5 mm in diameter were made using a puncturing tool (12.5 mm diameter). Texture profile analysis was done using the aforementioned Instron Universal Testing Machine (Model 3344 testing device, Norwood, MA, USA). Samples were compressed two times until the height was 75% of the original height. A compression probe was used with a 500 N load cell at a crosshead speed of 300 mm/min. Hardness (gf), springiness (mm), gumminess, chewiness, and cohesiveness were expressed as previously detailed [21].

#### Statistical analysis

One-way ANOVA followed by Duncan’s test was done using the aforementioned SPSS 20.0 statistical software (SPSS Inc., Chicago, IL, USA). Each experiment was performed in triplicate. A p-value <0.05 indicated a significant difference.

## RESULTS AND DISCUSSION

### Evaluation of myofibrillar protein gel

#### Cooking loss and gel strength

The CL and gel strength of MP mixed gels are shown in Table 1. The MP gels formulated with increased levels of curdlan decreased CL (p<0.05). During the formation of curdlan gel from a powder, free water moves into curdlan micelles and is bound by hydrogen bonding, resulting in improved water-binding capacity and a strong gel structure in the presence of a protein mixture including MP [22]. Funami et al [23] reported that heat-stable curdlan gel formed in meat products and hence its structures held water tightly. The gel strength progressively increased with the addition of the increased level of curdlan up to 1.0%. No differences in gel strength of MP get with 1.0% and 1.5% curdlan were observed. The gel of curdlan had much triple helices micelles with junction zone, which could be formed the gel [24]. Thus, forming the hydrogen bonded triple helix junction zones between curdlan and MP resulted in the increased gel strength. High-set curdlan gels, which were heated at 80°C, were progressed with hydrophobic bonding among curdlan molecules [22]. Curdlan became swollen during absorbing the water and gave the pressure among the gel matrix, thereby increasing the gel strength [25]. Gel strength can be increased by adding hydrocolloids such as konjac flour, which was a typical hydrocolloid for improving the gel strength in MP [26]. Since curdlan is also classified as a hydrocolloid, gel strength could be increased by adding curdlan on MP gels.

### Viscosity

**Figure 1:**
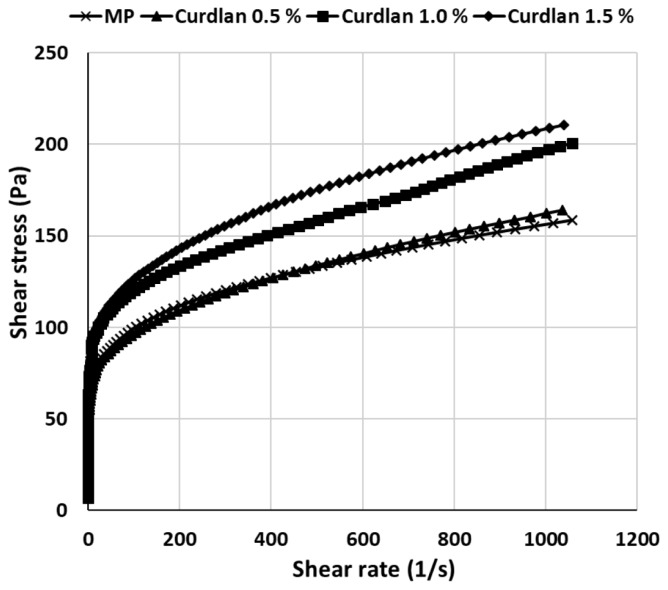
The viscosity of myofibrillar protein gel with various contents of curdlan.

The viscosity of MP mixtures with various levels of curdlan is shown in Figure 1. MP mixtures with 0.5% curdlan had low shear stress compared to those with higher concentrations of curdlan. MP mixture without curdlan showed similar value to those with 0.5% curdlan. However, shear stress of MP mixtures containing 1.0% and 1.5% curdlan was higher than that of curdlan-free control. Lo et al [27] also reported that apparent viscosity increased with increasing concentration of curdlan, which caused intermolecular cross-linking. These rigid three-dimensional structures had limited to mobile the polymer chain, indicating the increasing the shear stress by shear rate. Lee et al [28] reported that several carbohydrates combined well with protein and water, and modified the viscosity of the protein mixture. The viscosity of meatballs was also increased with increased levels of curdlan from 0% to 0.6% [13], which was attributed, at least in part, to the sticky properties of curdlan before heating. Water-dissolved curdlan had sticky properties, which was easy to interpenetrate among the three-dimensional meat protein structures, resulting in a bonding tightly and making a firm gel. As viscosity is related to mouthfeel during mastication, these results can attribute to the desirable texture when applied to meat products.

### Microstructure

**Figure 2:**
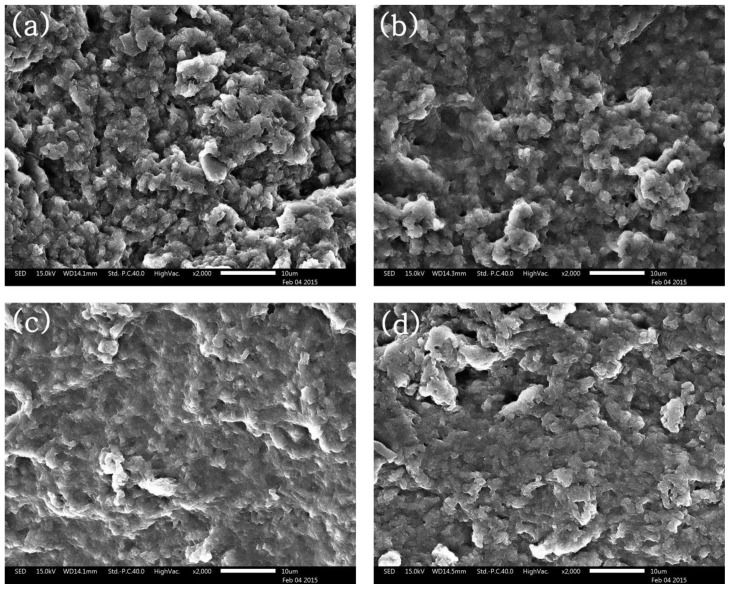
Scanning electron microscopy (×2,000) of myofibrillar protein with various contents of curdlan (a) control, (b) curdlan 0.5%, (c) curdlan 1.0%, (d) curdlan 1.5%.

Microstructures of MP gels with various levels of curdlan are shown in Figure 2. The MP gels generally adopted a globular structure. In the presence of curdlan, the MP microstructure appeared flat and less porous and curdlan might be occupied the MP pores, resulting in a smooth and swollen surface (Figure 2b, 2c, 2d). The MP gels containing 1.0% and 1.5% curdlan (Figure 2c, 2d) showed denser and filling-up appearance, as compared to the control (Figure 2a). Although curdlan didn’t affect the thermal stability of meat, it showed a well-mixed structure between curdlan and meat protein after heating [23]. Some of the pores were formed because the excess curdlan was not combined with MP and aggregated independently. This might be detrimental to the texture of model sausages formed with 1.5% curdlan. A globular structure was produced in the presence of 0.5% curdlan (Figure 2b), but the structure was less porous compared to the control (Figure 2a). These results agreed with those of Wu et al [25], who reported that curdlan molecules filled to the pores around protein matrix and it became a more compact and denser homogenous network through hydrogen bonding, resulting in a high gel strength and exemplary water retention. The natural state of curdlan granule had formed the doughnut-shaped structure, similar with starch structure [29]. Rearrangements of the dense structure have been attributed to curdlan and these structures might affect results of gel strength and CL [30].

### Characteristics of low-fat model sausage containing various levels of curdlan

#### pH, color, chemical composition, and textural properties

pH and color of LFSs are shown in Table 2. pH and lightness (L*) values did not differ among the treatments (p>0.05). However, redness (a*) and yellowness (b*) values of sausages with curdlan levels lower than 1.0% differ from those with 1.5% curdlan (p<0.05). These results indicated that curdlan level influences the color values. Hydrocolloids, such as carboxymethyl cellulose and sodium alginate, were reported to affect redness and yellowness values except for lightness [31]. Moisture, fat, and protein contents did not differ among the treatments (p> 0.05). Calliari et al [32] reported that chicken patties made with 5% polymer from *Agrobacterium radiobacter* k84 showed no differences in moisture and protein contents compared to polymer-free patties. The polymer of *Agrobacterium radiobacter* k84 is predominantly composed of minerals (40%) followed by carbohydrates (35%) and protein (15%) [33]. In this study, no differences in the chemical compositions were observed in model sausages with various curdlan levels. Cardoso et al [34] reported that carrageenan at 1.0% did not affect the chemical composition of cod frankfurter sausages.

Textural properties of LFSs are shown in Table 3. No differences in most textural characteristics of LFSs were observed (p>0.05), except for hardness values, which were different among the curdlan levels. The hardness of LFSs with 0.5% and 1.0% curdlan was higher than those without curdlan. By increasing contents of curdlan, the number of junction zone by hydrophobic bonding between curdlan and MP was increased and hence rigid structures appeared [15]. However, hardness was decreased with the addition of 1.5% curdlan, partially due to the disrupted protein-protein interaction (p<0.05), resulting in a more crumbled texture. Thus, excessive curdlan addition may disturb the cross-linking of the salt soluble protein, resulting in difficulties to form the strong gels [25]. Adding 1.0% amidated low methoxyl (ALM) pectin increased the firmness of restructured fish gels, but additional levels of ALM pectin disrupted the interaction with fish meat gels [35]. Adhesiveness, chewiness, and gumminess values of emulsified meatballs were increased with increasing levels of curdlan [13]. Kappa-carrageenan (0.5% to 2.0%) in low-fat meatballs increased hardness, adhesiveness, chewiness, and gumminess [36]. Montero et al [31] also reported that kappa-carrageenan and sodium alginate increased hardness, adhesiveness, and cohesiveness in blue whiting gels. Thus, an appropriate addition of hydrocolloid in the manufacture of meat products may improve the textural properties.

### Cooking loss and expressible moisture

**Figure 3:**
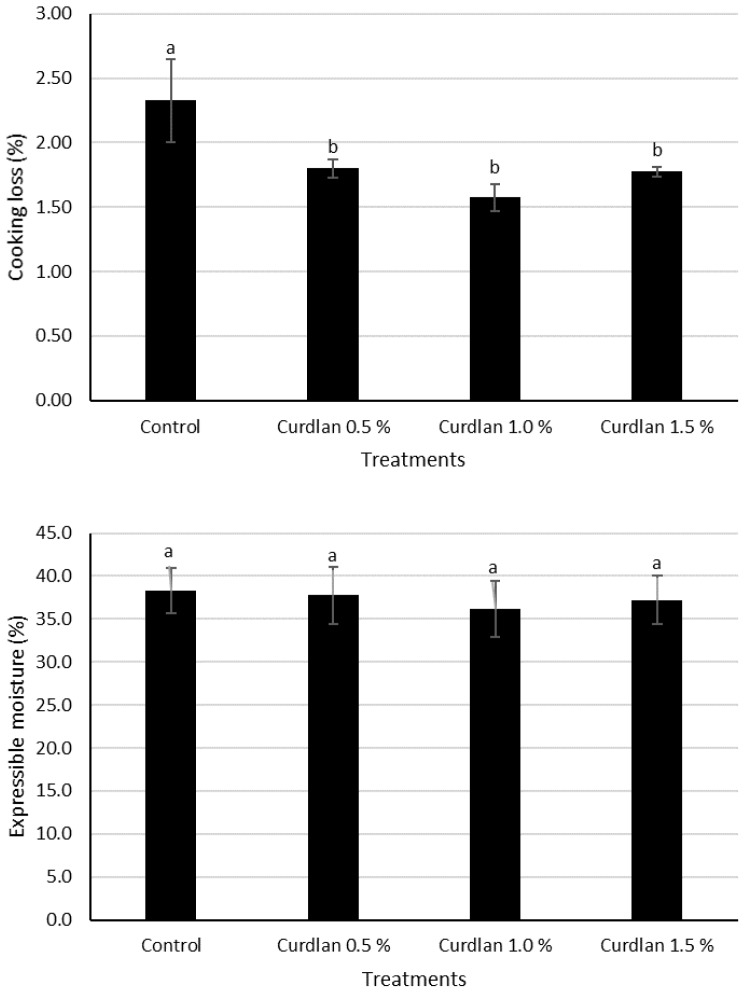
Cooking loss (%) and expressible moisture (%) of low-fat model sausages as affected by different levels of curdlan. ^a,b^ Means with diferent letters differ at p<0.05.

Figure 3 shows the result of CL (%) of low-fat model sausages with various curdlan contents. CL of LFSs made with the three levels of curdlan were lower than those without curdlan (p< 0.05). This result was supported by Kimura et al [37] who reported that the addition of curdlan in sausage increased the product yield. Funami and Nakao [16] observed that curdlan retained free and released water during cooking. This partially reflects the formation of hydrophobic interactions and hydrogen as affected by thermal stability [38]. Microfibrils, which are from curdlan, had broken the structure at 60°C and rearranged the structure at the higher temperature by hydrophobic interactions [24]. Funami et al [23] reported that released water during cooking was decreased as the levels of curdlan increased in minced pork gels. Curdlan was mixed well with meat proteins by chopping progress and the meat proteins were rearranged the curdlan-protein gel matrix during the heating. Increasing these cross-linking in meat gel system may affect to hold the water molecules strongly [25]. Thus, CL reduced by adding curdlan since curdlan had the high force to entrap the water within meat protein.

Although curdlan can hold water strongly among meat protein molecules, no differences in EM were observed among treatments, regardless of adding curdlan (p>0.05) (Figure 3). Generally, water-holding capacity of curdlan was reportedly 3–5 times higher than egg white in minced pork gel [23]. Hydration, hydrogen bonds, hydrophobic interactions, and Van der Waals forces between carbohydrates and proteins were thought to affect the water binding ability of protein gels [39]. In addition, the triple helix of curdlan was reinforced the interstitial water with hydrogen bond [22].

## CONCLUSION

The MP containing 1.0% and 1.5% curdlan increased the viscosity and gel strength, and CL was decreased in LFSs formulated with all three levels of curdlan. The addition of curdlan into MP or LFS resulted in a reduction in CL and therefore, curdlan improved overall quality characteristics of LFSs by retaining the water. Hardness also increased in low-fat model sausages with 0.5% and 1.0% curdlan. However, the excess addition of curdlan reduced the textural characteristics. Thus, 1.0% of curdlan is considered as an optimal level in improving the physicochemical properties of low-fat model sausages. Further study is needed to determine the mechanism of the negative effect of a high concentration (1.5%) of curdlan on LFSs.

## Figures and Tables

**Table 1:** Cooking loss and gel strength of myofibrillar protein gel with various content of curdlan

Items		Curdlan (%)			

		Control	0.5	1.0	1.5
Cooking loss (%)	Mean	8.46^a^	5.78^b^	4.07^c^	2.64^d^
	SD	0.25	0.24	0.87	0.25
Gel strength (gf)	Mean	260^c^	297^b^	354^a^	385^a^
	SD	18.0	9.83	4.41	17.2

SD, standard deviation.

a–d Means (n = 3) having same superscripts in a same row are not different (p>0.05).

**Table 2:** pH and color values of low-fat model sausages as affected by different levels of curdlan

Items		Curdlan (%)			

		Control	0.5	1.0	1.5
pH value	Mean	6.24^a^	6.23^a^	6.23^a^	6.22^a^
	SD	0.16	0.18	0.18	0.17
CIE L*	Mean	74.5^a^	73.5^a^	73.5^a^	74.6^a^
	SD	0.11	0.57	1.01	0.61
CIE a*	Mean	9.96^a^	10.3^a^	9.78^a^	8.95^b^
	SD	0.27	0.32	0.32	0.26
CIE b*	Mean	5.08^b^	5.36^b^	5.21^b^	6.34^a^
	SD	0.42	0.18	0.27	0.06

SD, standard deviation; CIE, Commission Internationale de l’Eclairage.

a,b Means (n = 3) having same superscripts in a same row are not different (p>0.05).

**Table 3:** Textural properties of low-fat model sausages as affected by different levels of curdlan

Items		Curdlan (%)			

		Control	0.5	1.0	1.5
Hardness (gf)	Mean	1,901^b^	2,251^a^	2,311^a^	1,764^b^
	SD	32.0	87.9	150	177
Springiness(mm)	Mean	6.68^a^	6.82^a^	6.60^a^	7.06^a^
	SD	1.32	0.41	1.01	0.64
Gumminess	Mean	14.9^a^	16.7^a^	18.3^a^	14.4^a^
	SD	0.68	0.98	3.04	2.63
Chewiness	Mean	98.9^a^	109^a^	118^a^	102^a^
	SD	27.4	2.21	30.2	27.4
Cohesiveness	Mean	7.77^a^	7.59^a^	7.78^a^	7.79^a^
	SD	0.47	0.55	0.37	0.25

SD, standard deviation.

a,b Means (n = 3) having same superscripts in a same row are not different (p>0.05).
